# Cognitive Function and Atrial Fibrillation: From the Strength of Relationship to the Dark Side of Prevention. Is There a Contribution from Sinus Rhythm Restoration and Maintenance?

**DOI:** 10.3390/medicina55090587

**Published:** 2019-09-13

**Authors:** Emanuele Gallinoro, Saverio D’Elia, Dario Prozzo, Michele Lioncino, Francesco Natale, Paolo Golino, Giovanni Cimmino

**Affiliations:** Department of Translational Medical Sciences, University of Campania “Luigi Vanvitelli”, 80131 Naples, Italy; e.gallinoro@gmail.com (E.G.); saveriodelia85@gmail.com (S.D.); dario.prozzo@gmail.com (D.P.); michelelioncino@icloud.com (M.L.); natalefrancesco@hotmail.com (F.N.); paolo.golino@unicampania.it (P.G.)

**Keywords:** atrial fibrillation, cognitive decline, anticoagulation, rhythm control, microbleeds, cerebral ischemia

## Abstract

Atrial fibrillation (AF) is the most common chronic cardiac arrhythmia with an increasing prevalence over time mainly because of population aging. It is well established that the presence of AF increases the risk of stroke, heart failure, sudden death, and cardiovascular morbidity. In the last two decades several reports have shown an association between AF and cognitive function, ranging from impairment to dementia. Ischemic stroke linked to AF is a well-known risk factor and predictor of cognitive decline. In this clinical scenario, the risk of stroke might be reduced by oral anticoagulation. However, recent data suggest that AF may be a predictor of cognitive impairment and dementia also in the absence of stroke. Cerebral hypoperfusion, reduced brain volume, microbleeds, white matter hyperintensity, neuroinflammation, and genetic factors have been considered as potential mechanisms involved in the pathogenesis of AF-related cognitive dysfunction. However, a cause-effect relationship remains still controversial. Consequently, no therapeutic strategies are available to prevent AF-related cognitive decline in stroke-free patients. This review will analyze the potential mechanisms leading to cognitive dysfunction in AF patients and examine the available data on the impact of a sinus rhythm restoration and maintenance strategy in reducing the risk of cognitive decline.

## 1. Introduction

Atrial fibrillation (AF) is a common chronic cardiac arrhythmia with an increasing prevalence over time mainly because of population aging, peaking at 10–17%incidence from the age of 80 years and older [1,2]. The presence of AF increases the risk of stroke up to five-fold [3], heart failure [4,5] and death [6,7]. Epidemiological evidence indicates an association between AF, cognitive impairment and dementia [8,9,10,11,12,13]. The great impact of this issue is demonstrated by the several articles that have been published only in the last 12 months from the present review paper by Heart Rhythm Associations [14] and others [10,11,12,15,16,17,18,19,20,21,22].Stroke-related AF is a well-known risk factor and predictor of cognitive impairment and dementia [23]. However, clinically recognized strokes represent only the tip of an iceberg. Some observations suggest that AF-induced brain ischemia and silent brain infarcts [24,25,26] detected by neuroimaging [27] are more frequent than clinical stroke and together with microinfarcts (beyond the power resolution of the conventional neuroimaging techniques) are associated with cognitive impairment and dementia [28,29]. Based on recent observations AF may also be a predictor of cognitive impairment and dementia in the absence of stroke [30,31]. Moreover, taking into account the different patterns of AF (paroxysmal, persistent, long-stand persistent, permanent, non-valvular, and incident [1,32]), it seems clear that the association between AF and cognitive function becomes more difficult to elucidate. In addition, a full understanding of the mechanisms by which AF may lead to cognitive impairment also in patients without any evidence of stroke remain not completely understood [33]. Cerebral hypoperfusion, chronic inflammation and endothelial dysfunction have been considered potentially involved in the pathogenesis of AF-cognitive impairment [11,20,34,35,36]. Currently, no therapeutic strategies are available to prevent cognitive dysfunction in stroke-free patients with AF; therefore, clarifying the potential underlying mechanisms of cognitive impairment in AF without stroke might be a critical issue.

This review, starting from the available literature, focuses on the relationship between AF and cognitive impairment, exploring both stroke and non-stroke related mechanisms that lead AF-patients to the development of progressive cognitive dysfunction. Moreover, the examination of the potential basic mechanisms provides an insight into the possible therapeutic implications. Finally, the potential benefit of a sinus rhythm restoration and maintenance strategy is explored.

## 2. Current “Views” on Atrial Fibrillation-Related Stroke

As reported by the majority of epidemiological studies the presence of AF implies up to five-fold increased risk of ischemic stroke [3,37,38], but the causal relationship of this correlation still remains not completely understood [33]. Moreover, this risk increases if other pathological conditions, such as hypertension [39], diabetes mellitus, valvular heart disease [6], heart failure [5], coronary heart disease [6,40], chronic kidney disease [6,32], inflammatory disorders [41,42], sleep apnea [43], and tobacco use [44] are present. To date some of these comorbidities are also included in the CHA2DS2-VASc score used to calculate the annual risk of stroke [1].

### 2.1. Possible Mechanisms of AF-Related Stroke

The current hypothesis postulates that uncoordinated myocytes activity could explain the impaired/loss of atrial contraction seen in AF patients, and the resulting blood stasis would cause the increased thromboembolic risk [33,45]. Despite a direct correlation between AF and stroke found in many studies, this is not consistent among all available data: according to some reports, the risk of embolic stroke seems not to be directly related to the duration of dysrhythmia [46,47,48,49,50]. This evidence seems to demonstrate the lack of a direct association between the burden of AF and the prevalence of stroke. Furthermore, it is important to note that a single brief episode of subclinical AF is associated with a 2-fold higher risk of stroke in older patients with vascular risk factors, whereas young and otherwise healthy patients with clinically apparent AF do not face a significantly increased stroke risk [51,52]. These data support the role of other concomitant risk factors apart from dysrhythmia in the determination of AF-related stroke. If AF causes thromboembolism, it should be specifically associated with embolic strokes [53]. However, almost 10% of patients with lacunar strokes have AF, and large-artery atherosclerosis is twice common in AF patients, suggesting a possible contribution from other factors [54]. Moreover, if dysrhythmia is the only cause of thromboembolism, maintaining a normal rhythm should eliminate stroke risk. However, in a meta-analysis of eight randomized clinical trials, a rhythm-control strategy had no effect on stroke risk (odds ratio, 0.99; 95% confidence interval, 0.76–1.30) [55], and it is unlikely that this result could reflect a failure to maintain sinus rhythm because rhythm-control strategies showed substantial success in maintaining normal sinus rhythm (odds ratio, 4.39; 95% confidence interval, 2.84–6.78). Atrial fibrillation coexists with other alterations, such as endothelial dysfunction [56], fibrosis [57], and mechanical dysfunction of left atrial appendage [58]. These factors have been associated to stroke. Some authors have proposed a novel up to date model of AF-related stroke, based on the severity of atrial cardiopathy rather than the duration of dysrhythmia [33]. According to this new hypothesis, AF and thromboembolism occur as separate downstream effects of atrial cardiopathy [33,38,59]. Briefly, this model highlights the interaction between systemic vascular risk factors, atrial substrate and rhythm suggesting that these factors with the aging finally result in atrial cardiopathy, thus increasing the risk to develop AF and consequently thromboembolism. The role of atrial cardiopathy in thrombogenesis should be considered similar to the post myocardial infarction and heart failure related ventricular cardiopathy. In both of these diseases, thromboembolism can occur even in the absence of dysrhythmia. Once developed, AF causes contractile dysfunction and stasis because of dysrhythmia, which further increases the risk of thromboembolism [37,45]. In addition, long-standing persistent AF (a pattern that lasts at least a year without interruption) [1] causes atrium remodeling, thereby worsening atrial cardiopathy and increasing thromboembolic risk even further. On the other hand, systemic risk factors participate to increase risk of stroke via non atrium-related mechanisms, such as in situ cerebral small-vessel occlusion, atherosclerosis of the large-artery, and ventricular systolic dysfunction [60]. Finally, once stroke occurs, AF risk may transiently increase because of autonomic changes and post-stroke inflammation [61].

### 2.2. AF and Stroke-Related Cognitive Impairment: The Visible Side of the “Moon”

The relationship between AF and cognitive impairment/dementia has been reported in several studies [11,15,16,17,19,20,62,63]. The large cross-sectional Rotterdam Study was one of the first pieces of evidence to describe this association [64]. Of the 6584 participants, 635 (9.6%) had cognitive impairment without dementia, whereas 4.2% were diagnosed with dementia. In 75% of the affected patients, the most common form of dementia was Alzheimer’s disease, whereas 15% had vascular dementia and 11% undefined dementia. Of the patients with Alzheimer’s disease, almost 20% had concomitant cerebrovascular disease. Dementia was reported to be up to 2-fold more common in patients with AF than in those without it. A significant positive association between cognitive impairment and AF was also described, but this association was weaker [64]. Stratification for sex showed that these findings were restricted to women and patients younger than 75 years old [64]. Furthermore, a systematic review including more than 77,000 patients with normal baseline cognitive function and not suffering an acute stroke, showed that AF significantly increases the risk of incident dementia (HR 1.42, CI 1.17–1.72, *p* = 0.002) [30]. Three meta-analyses have shown a higher risk of dementia in patients with AF who have a stroke (RR 2.43–2.70) [30,65,66]. The risk of incident dementia and cognitive decline was more modest in those without stroke at baseline than in patients with AF and previous history of stroke. AF patients have up to a 2-fold higher risk of silent or subclinical strokes than those without AF [28]. In AF patients, subclinical stroke has been clearly associated to long-term rates of cognitive dysfunction and dementia compared to patients who do not have a stroke, and there is a direct correlation between the impairment of cognitive function and the number of silent cerebral lesions at MRI [26,28].

Of note, patients with persistent AF (defined as at least seven days of arrhythmia that may or may not end on its own [1]) have a significantly higher number of lesions than those with paroxysmal pattern, in which irregular heartbeat may last anywhere from several seconds to a week, but usually ends spontaneously within 24 h [1] (41.1 ± 28.0 vs. 33.2 ± 22.8, *p* = 0.04) [28]. Cognitive performance, assessed by well-validated tests, was significantly worse in patients with persistent and paroxysmal AF than in controls (Repeatable Battery for the Assessment of Neuropsychological Status scores 82.9 ± 11.5, 86.2 ± 13.8, and 92.4 ± 15.4 points, respectively, *p* < 0.01) [28].

Many of the previously cited studies were limited by the short duration of the follow-up. The Atherosclerosis Risk in Communities, a prospective cohort study with a 20-year follow-up, showed that participants who developed incident AF (defined as the first occurrence of hospitalization with a primary discharge diagnosis of AF or ≥2 ambulatory visits for AF [32])had greater cognitive decline over 20 years, compared to participants who did not develop AF. The AF-related decline in the global score was 16% greater and was augmented after accounting for attrition. In addition, incident AF was associated with 23% higher risk of dementia. Although adjustment for prevalent and incident ischemic stroke attenuated the associations slightly, they remained significant [8]. AF-related cognitive impairment was characterized by a greater decline in cognitive tests associated to language and executive function rather than memory tests [8]. While Alzheimer’s disease is mainly characterized by memory deficits [67,68,69], AF shares with other vascular risk factors a preferential impairment of visuospatial ability [28]. The study by Gaita et al. [28] evaluated the distribution of silent cerebral lesions in patients with paroxysmal or persistent atrial fibrillation, reporting bilateral distribution with cortical and subcortical areas of silent cerebral ischemia. These lesions showed a frontal spotted pattern which is in contrast with the hippocampal and temporal lobe involvement of Alzheimer’s disease. The cardiac origin of the embolic particles was suggested by distribution and size of the embolic material. Emboli of cardiac origin are generally smaller than those due to atherothrombotic material and cause lesions widely distributed, on both sides, of the brain [70,71,72]. The cerebral MR pattern described in 50% and 67% of the patients with paroxysmal and persistent AF, respectively, was characterized by small, sharply demarcated lesions, often in clusters, with a bilateral distribution, prevalently in the frontal lobe, strongly supporting the non-atherothrombotic origin of the silent cerebral ischemia.

Finally, another cross-sectional study reported that AF was not associated to cognitive decline in patients without prevalent silent cerebral ischemia and/or subclinical cerebral infarct [73].

### 2.3. Effects of Anticoagulation Therapy

Despite the well-documented role of anticoagulants in cardioembolic stroke prevention [1,74], it is not clear if the risk of AF-related dementia can be significantly reduced by oral anticoagulation [11,16,75,76,77]. Prior studies have found that oral anticoagulation in stroke free patients was associated with dementia [78]. Intracranial hemorrhage has been considered the major concern with anticoagulation use, and the risk was higher in patients with leukoaraiosis (white matter changes) [79]. In warfarin-treated patients, the maintenance of an international ratio between 2 and 3 for most of the time-period (defined as time in therapeutic range [80]) is essential for stroke prevention [1]. It has been reported that chronic undercoagulation as well as overcoagulation might be linked to increased risk of cognitive impairment (HR 1.017 CI 1.007–1.027, *p* = 0.001 and HR 1.018 CI 1.006–1.031, *p* = 0.005; respectively) [81]. This trend was found significant only in younger patients (<80 year old), most probably because of a longer anticoagulation regimen overtime [81]. A retrospective study from a Swedish Patient Register showed lower incidence of dementia among patients with oral anticoagulation than patients without anticoagulants (1.14 vs. 1.78 per 100 patients/year at risk, *p* < 0.001) [82]. The use of anticoagulation at baseline was associated with 29% lower risk of dementia than in patients without anticoagulant drugs (HR 0.71, 95% confidence intervals 0.68–0.74 and 48% lower risk analyzed on treatment (HR 0.52, 95% CI 0.50–055) [82].

In the last decade, the use of novel anticoagulants (direct inhibitor of coagulation factor Xa or thrombin, named DOACs)that do not require lab monitoring has greatly improved the prevention of AF-related cardiac embolism [83], even in elderly [84,85] and in patients undergoing cardioversion [86,87], because of a better compliance and a uniform time in therapeutic range [59,83,88]. Based on the current literature, risk of undercoagulation as well as overcoagulation should be overcome [83]. The risk of dementia appeared to be lower with DOACs (HR 0.48, 95% CI 0.40–0.58) than warfarin (HR 0.62, 95% CI 0.60–0.64), but direct comparison showed no significant differences [76,82,89]. A recent meta-analysis including 471,057 AF patients under oral anticoagulants has shown that anticoagulation was associated with a significant reduction in cognitive impairment [90]. Moreover, comparison of DOACs with warfarin-based treatment showed that the novel agents-based group has a significantly lower occurrence of dementia with an increased risk of bleeding in warfarin group [89,90]. Furthermore, in the DOAC-treated group, a low combined risk of dementia and stroke was also reported [89].

However, pre-specified blind and randomized clinical trials are warranted to verify the role of oral anticoagulation in the prevention of dementia and resolved the current controversies. Actually, the Blinded Randomized Trial of Anticoagulation to Prevent Ischemic Stroke and Neurocognitive Impairment in AF (BRAIN-AF) (NCT02387229) is ongoing [91]. It is enrolling patients with non-valvular AF (defined as arrhythmia that is not caused by any moderate to severe heart valve disease [1]) that will be screened for dementia prior to randomization by mini-mental state examination and other tests. The efficacy and safety of rivaroxaban 15 mg will be evaluated for stroke reduction, transient ischemic attack and neurocognitive decline [91]. Another trial entitled “Impact of Anticoagulation Therapy on the Cognitive Decline and Dementia in Patients with Non-Valvular Atrial Fibrillation (CAF—NCT03061006)”, randomized, will compare the use of dabigatran vs. warfarin in 120 AF patients to assess the cognitive decline through neurological examination and cognitive testing [77].

Based on the current evidence, it has been suggested that DOACs would be a better choice for prevention of dementia than warfarin [92]. The lower rate of intracerebral bleeding has been suggested to be one possible mechanisms involved in this protective effect [92], but further studies are needed in order to investigate the role of DOACs in prevention of AF-related cognitive impairment and in the definition of a cause-effect relationship rather than a simple epidemiologic association.

## 3. Atrial Fibrillation and Non-Stroke-Related Cognitive Decline: The Submerged Part of the Iceberg?

In the last decade, new evidence supported the role of AF as independent risk factor for cognitive impairment and dementia even in patients with no history of stroke as assessed by two meta-analysis including large samples of patients [30,66] as well as by a perspective post-hoc analysis of two randomized clinical trials: the ONTARGET and the TRASCEND [31]. Large longitudinal studies also provided data supporting this association. Chen et al. [8] analyzed the results from a cohort of more than 12,000 patients enrolled in the ARIC study and evaluated the association of incident AF with 20-year change in cognitive performance considering the incidence of dementia and the cognitive decline: In conclusion, AF increased the risk of cognitive impairment and dementia independently from ischemic stroke (global cognitive Z score = 0.115, 95% confidence interval, 0.014–0.215) [8]. Similarly, De Bruijn et al. [62] evaluated the association of incident and prevalent AF and incident dementia in 6514 dementia-free participants in the prospective population-based Rotterdam Study over a 20-year follow-up period showing that prevalent and incident AF increases the risk of dementia (HR 1.33; 1.02–1.7 for prevalent AF and 1.23 (0.98–1.56) for incident AF, 95% CI) especially in younger patients (<67 year old) and in those with longer duration of AF [62].

### Linking Mechanisms of AF to Cognitive Dysfunction in Stroke-Free Patients

Despite this epidemiological evidence, the pathophysiological mechanisms correlating AF and cognitive dysfunction in stroke-free patients are not completely elucidated. It is widely known that microbleeds, which are often the result of hypertensive vasculopathy/fibrohyalinosis and cerebral amyloid angiopathy, are associated with cognitive impairment [93]. In some AF patients, anticoagulation therapy may favor the occurrence of microbleeds, a condition that, at least in part, could explain the progressive cognitive impairment observed in AF [94]. However, to date there is a lack of studies about microbleeds and cognitive function in stroke-free patients affected by AF.

**Figure 1:**
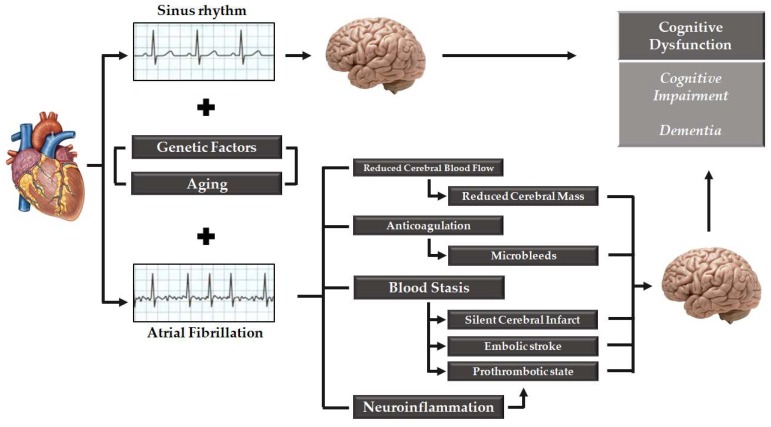
View of the mechanisms involved in cognitive dysfunction in patients affected by atrial fibrillation.

Brain white matter hyperintensity detected by MRI evaluation are associated with AF and poor cognitive performance [95]. However, the pathogenesis of white matter hyperintensity remains not completely understood, and its occurrence may be associated to cerebral hypoperfusion, arterial hypertension, aging, and cerebrovascular disease [96]. Neuroinflammation may be another possible explanation of the cognitive impairment in AF [41,97]. Several inflammatory markers are elevated in patients with AF such as C-reactive protein, tumor necrosis factor-α, interleukin-2, interleukin-6, and interleukin-8 and they may trigger cerebral micro-infarction and subsequent cognitive dysfunction by inducing a prothrombotic state through endothelial activation/damage, production of tissue factor from monocytes, increased platelet activation, and increased expression of fibrinogen [98]. Lappegard et al. demonstrated that anti-inflammatory therapy through intensive lipid-lowering treatment with 40 mg atorvastatin and 10 mg ezetimibe can modify the deterioration of neurocognitive function, and the loss of volume in certain cerebral areas in older patients with AF [99]. Reduced brain volume has been considered another potential risk factor linking AF and cognitive function. In a cross-sectional analysis of 4252 participants without dementia, AF was associated with a lower volume of gray and white matter (*p* < 0.001 and *p* = 0.008, respectively) [100]. The association was reported to be even stronger in patients with persistent AF compared to paroxysmal AF [100]. A smaller hippocampal volume, evaluated by structural MRI, has been associated with neurocognitive decline and progression towards Alzheimer disease in patients with mild cognitive impairment [101,102]. In a cross-sectional analysis, led by Knecht et al. on 122 patients, patients with AF without stroke showed worsening in tasks of learning and memory (*p* < 0.01) as well as attention and executive functions (*p* < 0.01) compared to subjects without AF; corresponding to the memory impairment, hippocampal volume was reduced in AF patients [103]. Genetic risk factors predisposing to dementia and cognitive impairment have been extensively studied but whether these factors may link AF and cognitive dysfunction is not well established. In the study by Rollo et al. 112 Caucasian patients with AF and dementia were matched 1:1 with patients with AF and without dementia resulting in an association between PITX2 loci, rs2200733, and dementia (OR = 2.15, *p* = 0.008) [104]. However further studies are warranted to confirm these results and clarify the role of genetic factors which may influence development of cognitive dysfunction in AF patient. Most of the mechanisms involved in AF-related cognitive dysfunction are summarized in Figure 1

## 4. Rhythm Control Strategy and Cognitive Impairment: The Dark Side of the Prevention?

The management of AF patients has been the subject of intensive investigations especially in the 1980s and early 1990s. It is well documented that compared with patients in sinus rhythm, the development of AF increases the risk of stroke [3] and worsen cardiovascular outcomes in patients with heart failure (HF) [5]. Nevertheless, it has to be taken into account that AF is a marker of more severe disease, and thus, evaluation of the coexisting comorbidities and their respective contribution to the worsening of long-term prognosis in AF patients should be carefully evaluated [5,32,39].

It seemed to be logical that restoration and maintenance of sinus rhythm might improve cardiovascular outcome. However, the analysis of the large clinical trials evaluating the impact of either rate or rhythm control strategy on mortality or combined end point of mortality and morbidity have demonstrated no benefits [105,106,107,108,109], resulting in a rethinking of the appropriate way in which to treat a patient with AF when the therapeutic options include both strategies, rate or rhythm control [110]. To date, this question still remains a matter of debate [111,112].

### 4.1. AF, Cerebral Blood Flow, and Possible Contribution to Cognitive Impairment

Among older adults, lower cardiac index is associated with reduced cerebral blood flow in the cerebral gray matter, especially in lobes [113]. This mechanism seems to be associated with incident dementia and Alzheimer’s disease [114]. It is well documented that AF can reduce cardiac output [115,116]. Thus, by reducing cardiac output, AF could induce chronic brain hypoperfusion, which could be linked to AF-related cognitive impairment [104]. However, despite the association between AF and a reduction of almost 20% of the total cardiac output, cerebral blood flow has been reported by some evidence to be substantially unchanged, due to autoregulation mechanisms [117].

Currently, cardioversion of AF can be easily and safely performed because of DOACs [87,118,119,120]. A recent study has evaluated the impact of AF and sinus rhythm on cerebral blood perfusion [121] reporting that mean cerebral flow rates in AF and sinus rhythm are similar, even considering cerebral autoregulation (but not other associated pathologies). The authors concluded that a well-functioning cerebral autoregulating system is able to ensure a normal cerebral blood flow both during AF and sinus rhythm [121]. These findings are apparently in contrast with the hypothesis that AF is associated to chronic brain hypoperfusion. Flow variability is higher in AF compared to sinus rhythm, with a peak at arteriolar and capillary levels, thus resulting in local hypoperfusion [121]. Taking in to account these observations, the hemodynamic cerebral effect of AF could be a relevant mechanism into the genesis of AF-related cognitive impairment/dementia. In fact, deep white matter could undergo an ischemic damage because of two possible mechanisms: (1) the transient hypoperfusion as indicated above or (2) as a consequence of being exposed to transient hypertensive events (by arteriolosclerosis and capillary loss/bleeding), laying the basis for a potential AF-related vascular subcortical dementia [122]. On this matter, a cross-sectional study evaluating an unselected elderly cohort showed that AF is associated with decreased total cerebral blood flow compared to those who were in sinus rhythm, assessed by on phase-perfusion MRI and a reduction in total brain volume [123]. Brain perfusion was lowest in the persistent AF group compared to the paroxysmal AF group (46.4 mL/100 g/min vs. 50.9 mL/100 g/min; *p* < 0.05) and those with no AF (52.8 mL/100 g/min; *p* < 0.001) [123]. Although the hemodynamic effects on brain are complex, evidence suggests that a decreased cerebral blood flow may play a role in reducing brain volume and inducing decline in cognitive function seen in AF patients [100]. Of note, patients with paroxysmal AF who were in sinus rhythm at the time of MRI had higher cerebral blood flow and higher relative brain volume compared to those with permanent AF suggesting that as longer is the persistence in AF as low is the cerebral perfusion [123]. There is also evidence supporting that both cerebral blood flow and brain perfusion, assessed by phase-contrast MRI, improve after cardioversion [124,125]. This evidence further supports the hypothesis that also the time-period in AF may influence the risk to develop cognitive impairment [17].

### 4.2. Rhythm Maintenance Strategy: Do We Have Supporting Evidence?

Data regarding the impact of rate control in AF and the incidence of cognitive dysfunction and dementia are still not conclusive. In the observational study from Bunch et al. the impact of effective AF ablation on the risk of cognitive decline and dementia was evaluated [126]. A total of 37,908 participants were enrolled from the large ongoing prospective Intermountain AF study and divided in three cohorts: (1) patients who underwent AF ablation (4212); (2) age/gender-matched controls with AF (no ablation, 16,848); and (3) age/gender-matched controls without AF (16,848). These cohorts were followed for at least 3 years. Authors reported a significant reduction of Alzheimer’s dementia in AF-ablated patients (0.2%) compared to AF patients who did not underwent ablation (0.9%) and patients without AF (0.5%) [126]. Patients treated with catheter ablation for AF have long-term rates of death, stroke, and dementia similar to patients without AF. Other types of dementia occurred in 0.4% of the AF-ablated patients compared to 1.9% of the AF patients not undergone ablation and 0.7% of the control patients [126]. A recent report by Damanti et al. [22] seems to shed more light on this issue, supporting the protective role of rhythm control strategy on cognitive function. Specifically, in their retrospective analysis, 1082 individuals aged 65 and older with AF before hospital admission (for any cause) were enrolled. Logistic regression evaluation adjusted for age, sex, education, antithrombotic therapy, and comorbidities found that the rhythm control strategy and education were associated with less probability of cognitive impairment.

In Table 1 are reported the published data on the putative role of sinus rhythm of cognitive function. However, based on this available literature, the impact of sinus rhythm restoration and maintenance on the cognitive decline should be further investigated in pre-specified randomized clinical trials. The major limitation for such investigation will be the need of many thousands of patients to enroll and a longer follow-up to be run for at least 10 years. Therefore, because it is unlikely that such trials will ever be funded, the real role of sinus rhythm restoration and maintenance will remain a pathophysiological based strategy but with a dark side in prevention of cognitive impairment.

## 5. Hypertension: A Non-Stroke-Related Mechanism of Cognitive Decline in Atrial Fibrillation?

While hypertension is a known risk factor for AF [39,127] the contribution of hypertension on cognitive decline in combination with AF is not well defined in literature. Among patients with established AF, hypertension is present in ≈60% to 80% of individuals [128]. In the ARIC study (Atherosclerosis Risk in Communities), hypertension was the main contributor to the burden of AF, explaining ≈20% of new onset of dysrhythmia [129]. However, the effect of an intensive control of blood pressure on the risk of new onset of AF in hypertensive patients remains unclear [39]. As reported by the post-hoc analysis of the ONTARGET and TRANSCEND trials the association of AF and cognitive impairment was independent of treatment with antihypertensive drugs [31]. However, other studies seem to confirm the contribution of hypertension in the cognitive impairment [130]. In a 5-year longitudinal study of 353 community-dwelling persons, mean age 72 years, increased blood pressure variability was associated with poorer cognitive function [131]. In addition, another study involving 1373 French participants aged 59 to 71 years, the risk of cognitive impairment at 4-year assessment was increased 2.8-fold in hypertensive patients [132]. It has been reported that because of hypertension, the structure and function of cerebral blood vessels is impaired, leading to ischemic damage of white matter regions that is critical for cognitive function [130]. However, whether hypertension treatment might reduce the risk for AF and the associated cognitive impairment remains unclear [39]. It has to be considered that in AF patients, the improvement of cognitive function related to the restoration and maintenance of sinus rhythm might be partially lost by an uncontrolled hypertensive status [130]. To date, despite the lack of evidence in the relationship between hypertension treatment and cognitive impairment, the uncontrolled blood pressure remains a major epidemiological contributor to cardiovascular disease and neurological disorders, and thus, an aggressive approach, as suggested by the current guidelines [133], is highly recommended.

## 6. Discussion

Several studies have evaluated the association between AF and cognitive dysfunction, ranging from cognitive impairment to dementia. Although the strongest evidence supports the role of stroke as the principal risk factor for cognitive impairment, it has been also established that AF is a risk factor for cognitive dysfunction independently from stroke [8,63]. Various mechanisms linked to cognitive impairment in AF patients, apart from stroke, have been discussed, involving microbleeds, white matter hyperintensity, neuroinflammation, reduced brain volume, cerebral hypoperfusion and genetic factors (as shown in Figure 1). However, a clear cause-effect relationship between these putative mechanisms and cognitive dysfunction is still controversial. Some of these mechanisms (i.e., microbleeds, reduced brain volume and cerebral hypoperfusion) might be linked to a common pathophysiological substrate which might be the impact of rhythm control over a rate control strategy on brain damage. First, since AF, through the loss of atrial systole contribution in left ventricle filling, reduces cardiac output and cerebral blood flow, restoration of sinus rhythm could improve brain perfusion, thus resulting in better cognitive outcome. Second, cerebral hypo-perfusion might be related to beat-to-beat variation in stroke volume in AF [100,123]. Decreased cerebral perfusion has been associated with a reduction in both grey and white matter although the effect may be greater on the grey matter due to higher metabolic demand [123]. However, reduction in grey matter is heterogeneous in the brain, since some areas appears to have higher vulnerability to cerebral hypoperfusion [134]. In some studies, a correlation between volume reduction in specified brain area (such as hippocampus) in AF patients and neurocognitive impairment and dementia has been reported [103]. Other investigations have linked the cerebral hypoperfusion AF-related and the reduction of brain volume [123]. Of note, the reduction in brain volume, especially in grey matter volumes of temporal and hippocampal areas, has been clearly associated with the risk of dementia [135]. Moreover, also white matter hyperintensity, linked with AF and cognitive impairment, may lead to cerebral hypoperfusion [95]. Taken together, these data are consistent with the “hemodynamic hypothesis” of AF related dementia and, as a consequence, restoration and maintenance of sinus rhythm might be associated to an improved brain perfusion, thus potentially avoiding most of the “risk factors” associated to cognitive impairment.

Further evidence linking AF and cognitive impairment comes from the SWISS-AF trial, a prospective multicenter national cohort study of 2400 patients across 13 sites in Switzerland [136]. In this study, patients with documented AF underwent to extensive phenotyping and genotyping, repeated assessment of cognitive functions, quality of life, disability, electrocardiography and cerebral magnetic resonance imaging. Authors reported that four in ten patients with AF but no history of stroke or transient ischemic attack had clinically unrecognized ‘silent’ brain lesions or other structural brain abnormalities such as white matter lesions, microinfarctions or microbleeds that could serve a substrate for the cognitive decline [136].

The question whether a rapid restoration of sinus rhythm in AF patients may reduce the risk for AF-related cognitive impairment remains a matter of debate. Further studies specifically designed to compare patients in a rhythm control intervention vs. rate control strategy are warranted to define the role of these two therapeutic approaches in the cognitive outcome overtime. Moreover, evaluation over time of specific parameters, such as cerebral blood flow, brain mass, neuroinflammation and others, will be of great importance to identify the contributing factors involved in cognitive impairment in stroke-free patients and the putative role of a rhythm restoring/rate control strategy in the prevention of cerebral damage. However, the major limitation in testing the effect of sinus rhythm restoration and maintenance in preventing cognitive decline and dementia is the need of larger (with many thousands of patients to enroll) and longer (to run for more than 10 years) prospective randomized trials. Therefore, it is unlikely that such trials might be ever funded.

## 7. Conclusions

AF is an independent predictor for cognitive dysfunction ranging from cognitive impairment to dementia. Apart from stroke and ischemic substrate, other mechanisms have been studied to explain the risk for cognitive dysfunction in AF patients. By the pathophysiological point of view, restoration and maintenance of sinus rhythm might represent an additional intervention to reverse some of the pathological alterations that serve as substrate for cognitive impairment, thus with potential effect in prevention. However, this aspect remains to be better supported by stronger evidence.

## Figures and Tables

**Table 1:** Studies comparing impact of sinus rhythm vs. atrial fibrillation on cognitive function.

Author	Type of Study (N. of Patients)	Design and Aim of the Study	Study Results
Damanti [22]	Retrospective Study 1802 individuals	Evaluation of cognitive performance using the Short Blessed Test according to rhythm and rate control strategy, antithrombotic therapy, age, education, and comorbidities.	In the absence of optimal anticoagulation, a rhythm control strategy is associated with lower probability of cognitive impairment.
Anselmino [108]	Experimental model	Two coupled lumped-parameter models (systemic and cerebrovascular circulations, respectively) were used to simulate sinus rhythm (SR) and AF. For each simulation 5000 cardiac cycles were analyzed and cerebral hemodynamic parameters were calculated	Higher cerebral flow variabiality in AF rather than SR may lead to subcortical vascular dementia
Gardarsdottir [110]	Cross-sectional study 2291 patients	Blood flow in the cervical arteries was measured with phase contrast MRI and brain perfusion. Individuals were divided into three groups at the time of the MRI: persistent AF, paroxysmal AF, and no history of AF	Reduced Brain perfusion in persistent AF compared to paroxysmal AF and SR. Patients with persistent AF had the smallest relative brain volumes when compared with the paroxysmal AF group and to those with no history of AF.
Gardarsdottir [111]	Observational study 26 patients	To measure cerebral blood flow (CBF) and brain perfusion (BP) with phase-contrast (PC) magnetic resonance imaging (MRI) and arterial spin labelling (ASL) MRI in patients with AF before and after cardioversion.	Cerebral blood flow and brain perfusion both improved after cardioversion to SR as opposed when patients continued to be in AF
Bunch [113]	Observational study 37,908 patients	Three groups of patients were enrolled: those who underwent AF ablation were compared to age/gender matched controls with AF (no ablation) and age/gender matched controls without AF. Impact of effective AF ablation on the risk of cognitive impairment and dementia was evaluated.	AF ablation patients have a significantly lower risk of death, stroke, and dementia in comparison to AF patients without ablation.
